# Prediction of total hospital expenses of patients undergoing breast cancer surgery in Shanghai, China by comparing three models

**DOI:** 10.1186/s12913-021-07334-y

**Published:** 2021-12-13

**Authors:** Minjie Chen, Xiaopin Wu, Jidong Zhang, Enhong Dong

**Affiliations:** 1 Renji Hospital, Shanghai Jiao Tong University School of Medicine, No. 160 Pujian Road, Shanghai, 200127 People’s Republic of China; 2 School of Nursing and Health Management, Shanghai university of medicine and health sciences, No.279 Zhouzhu Road, Shanghai, 210318 China

## Abstract

**Background:**

Breast cancer imposes a considerable burden on both the health care system and society, and becomes increasingly severe among women in China. To reduce the economic burden of this disease is crucial for patients undergoing the breast cancer surgery, hospital managers, and medical insurance providers. However, few studies have evidenced the prediction of the total hospital expenses (THE) for breast cancer surgery. The aim of the study is to predict THE for breast cancer surgery and identify the main influencing factors.

**Methods:**

Data were retrieved from the first page of medical records of 3699 patients undergoing breast cancer surgery in one tertiary hospital from 2017 to 2018. Multiple liner regression (MLR), artificial neural networks (ANNs), and classification and regression tree (CART) were constructed and compared.

**Results:**

The dataset from 3699 patients were randomly divided into training and test sets at a 70:30 ratio (2599 and 1100 records, respectively). The average total hospital expenses were 12520.54 ± 7844.88 ¥ (US$ 1929.20 ± 1208.11). MLR results revealed six factors to be significantly associated with THE: age, LOS, type of disease, having medical insurance, minimally invasive surgery, and receiving general anesthesia. After comparing three models, ANNs was the best model to predict THEs in patients undergoing breast cancer surgery, and its strong predictive performance was also validated.

**Conclusions:**

To reduce the THEs, more attention should be paid to related factors of LOS, major and minimally invasive surgeries, and general anesthesia for these patient groups undergoing breast cancer surgery. This may reduce the information asymmetry between doctors and patients and provide more reliable cost, practical inpatient medical consumption standards and reimbursement standards reference for patients, hospital managers, and medical insurance providers ,respectively.

**Supplementary Information:**

The online version contains supplementary material available at 10.1186/s12913-021-07334-y.

## Introduction

Breast cancer comprises different histopathological entities, which vary from benign tumors to highly aggressive cancers. It imposes a considerable burden on both the health care system and society [1, 2]. It is now the most common cancer in women in China as well as in many Western countries [3]. The burden of disease caused by breast cancer is becoming increasingly severe in China, especially in urban areas [4]. Over 50% of women in China experience benign breast problems [5–7]. However, the lack of adequate high-level evidence related to breast cancer causes its management to be based on an individual physician’s experience or training [8]. Furthermore, with the development of image-guided localization and expansion of the applications of ultrasonography, the accurate detection of suspicious nodules and lesions has increased; consequently, this has increased the chances of breast cancer surgeons to encounter new or challenging high-risk lesions. In general, patients with high-risk lesions are managed with routine monitoring and prevention, with risk-reduction surgery offered in some cases. Accordingly, prediction of the total hospital expenses for patients undergoing breast cancer surgery is essential as it may guide medical resource allocation and health policy decision-making in low- and middle-income countries, such as China.

For hospitalization cost analysis, traditional statistical methods, such as multiple linear regression(MLR) [9], logistic regression [10], and Cox regression models [11], have been generally used. These methods, however, have strict requirements for data, including data normality and independence. In recent years, data mining techniques, such as decision trees, neural networks, and support vector machines, have been increasingly adopted in the medical field. These techniques derive internal relations and rules from the sample data and then help predict and analyze unknown or unobservable data. Artificial neural network (ANN) and classification and regression tree (CART) models have been used for hospitalization cost prediction for various diseases, including genitourinary diseases [12], colorectal cancer [13], and gastric cancer [14]. However, few studies have evidenced the prediction of the total hospital expenses for breast cancer surgery. Therefore, we used a model mixed with ANNs and CART to predict total hospital expenses for breast cancer surgery and analyze the main influencing factors. Our findings may contribute to standardizing medical treatment procedures, reducing the wastage of health care resources, and formulating a reasonable security system for medical institutions and insurance companies.

## Methods

### Data sources

Data were retrieved from the first page of medical records of 3699 patients undergoing breast cancer surgery in Renji Hospital, Shanghai Jiao Tong University School of Medicine from January 1st, 2017, to June 30th, 2018. Renji Hospital is one of the largest tertiary general hospitals in Shanghai, with more than 12,900 admissions and 4.3 million outpatients/emergency visits in 2017. The first page of the medical record reflects the basic characteristics of patients and relevant information about hospitalization. It mainly includes the basic characteristics of patients, such as name, sex, age, admission diagnosis, discharge diagnosis, inpatient ward, date of surgery, type of surgery, and expenses during hospitalization.

### Outcome and predictive variables

#### Outcome variable: total hospital expenses

The outcome variable of total hospital expenses refers to the total payment that the patient’s primary insurance carrier provided to the hospital during the time from patient admission to discharge, including medical procedures, lab tests, supplies, and medications.

#### Predictive factors

To our best knowledge, the predictive factors extracted from the patients’ medical records were categorized into patient-related and hospital-related factors. Among them, the former predictive factors included age, sex, type of disease, length of hospital stay (LOS), having medical insurance, classes of surgery, minimally invasive surgery, day surgery, and receiving general anesthesia. For the convenience of analysis, these factors were standardized to quantitative values as dichotomized or categorical variables (Table 1).

The term major surgery was used to refer to mastectomy, regional lymph node dissection, and oncoplastic surgery. Minor surgery included breast-conserving surgery, quadrantectomy, lumpectomy, (sentinel) lymph node biopsy, nipple biopsy, biopsy of chest wall or skin lesion, and drainage of breast abscess. Minimally invasive surgery included minimally invasive mass resection or ultrasound-guided vacuum-assisted breast biopsy.

Notedly, since the final breast cancer staging was identified for each patient combining the pre-surgery evaluation, surgical findings, and most importantly the pathological result of the specimens removed in the surgery, which usually took an average of 2 weeks to get during that time. Based on the average length of stay being 4.86 ± 3.83 days, most of the patients in our study have already been discharged before getting the final staging confirmed. Thus, we assumed that the breast cancer staging was not decisive of the expenses of the surgical hospitalization we examined in this study and was not included in the constructed model.

#### Data analysis

The Kolmogorov–Smirnov test was conducted to assess the normal distribution of the total hospitalization expenses data. Three separate models were constructed: MLR, Backpropagation – Artificial Neural Network (BP-ANN), and CART. Before constructing the models, the association between preoperative factors and total hospital expenses was evaluated with univariable analysis by using Student’s *t* test or Spearman test for assessing continuous variables and the chi-square test or Mann–Whitney test for assessing categorical variables. SPSS v20.0 (IBM Corp, Armonk, NY, USA) was used for all statistical analyses. *P* < 0.05 (two-tailed) was considered statistically significant for all tests.

#### Model construction

Regression analysis has been widely used to predict patient outcomes. In this study, factors that were significant in the single-variant covariance test were included in a step-wise multiple linear regression model. Considering the nonnormal distribution of the total hospital expenses data, we logarithmized the values and constructed a multiple linear regression as follows:

1 $$Log(Y)={\beta}_0+{\beta}_1{X}_1+{\beta}_2{X}_2+\dots +{\beta}_m{X}_m+e$$

In (1), Y indicates the predicted value of the total hospital expenses; B_0_ represents the log(Y) − inte*rcept* (*value of* log(*Y*)*when all other parameters are set to* 0); *X*1, *X*2, …, *Xm represent the aforementioned significant independent factors*; *B*1*X*1 (*BmXm*) *indicates the regression coefficient B*1 (*Bm*) *of the first* (*last*) *independent variable X*1 (*Xm*); *and e indicates model error .*

A CART is a data mining method and a subset of the decision tree algorithm. Decision tree algorithms are often straightforward with high interpretability; they use a hierarchical tree structure for classification and regression. A CART algorithm does not need to consider any previous assumptions about the relationship and potential co-multicolinearity among variables. The Gini impurity measure was used for categorical targets, and using this method the data set is split into uniform domains based on yes/no answers regarding the predictor values, leading to the creation of a binary tree. The developed tree is called a classification or regression tree depending on whether the dependent variable is qualitative or quantitative, respectively [15]. In this study, all factors were inputted into models as independent variables.

ANNs are mathematical models based on interconnected groups of artificial neurons. They consider nonlinear relationships between input data, which are not always identified on traditional analysis. ANNs have several advantages, such as self-learning, adaptability, and robustness of massive parallelism. They generally contain three layers: input, hidden, and output layers. A multilayer perceptron (MLP) is a subtype of ANN comprising one or more hidden layers containing computation nodes, with a high capability of universal approximations; it has therefore been extensively used for modeling nonlinear and complex processes of the real world. The most widely used and effective algorithm for training an MLP network is the back-propagation (BP) algorithm [15–19]. The transfer function of the sigmoid was used in the ANN model with one hidden layer and three hidden nodes. The training algorithm is as follows:

2 $$f(x)=1/\left(1+{e}^{-x}\right)$$

We used numerical predictor nodes to automatically create and compare default models for continuous numerical outcomes. Default values were initially set in numerical predictor nodes. Given the methodological controversies and disagreements in current academic circles on how to divide the total hospital expenses data into different levels (e.g., high and low levels using K-means clustering or averaging method), we considered total hospital expenses, which is a continuous variable, as just an output variable without dividing it into categorical intervals. The input variables were selected in the prediction models according to the results of correlation analysis based on their *P* values. Moreover, we randomly divided the dataset into training and test sets at a 70:30 ratio (2599 and 1100 records, respectively). Both CART and ANN decision models were constructed using SPSS Modeler v18.0.

#### Model comparison

To test the performance of prediction capacity of models developed in this study, we used receiver operating characteristics curve (ROC) and calculated the area under receiver operating characteristics curve (AUC) scores to measure the discrimination of the prediction model. AUC represents the probability of correctly selecting variables into the model. An AUC score more than 0.50 (the diagonal) means the ability of model to correctly classify is good. The larger the number, the better the result, and 1.00 is perfect. Additionally, to compare the three models, some other critical performance indicators were used: model specificity(TNR), sensitivity(TPR), positive predictive value (PPV), negative predictive value (NPV), false-positive rate (FPR), false-negative rate (FNR), F1-score , Matthew’s Correlation Coefficient (MCC) and overall accuracy (OA).

#### Model validation

The best one of these three models was adopted for the sample. The aforementioned indicators were used to determine whether the model had good predictability.

## Results

### Basic characteristics of the enrolled patients

The average age of the 3699 breast cancer surgery patients was 45.58 ± 14.75 years. Their average LOS was 4.86 ± 3.83 days. Among them, 1195 patients paid the hospital expenses themselves and 2504 paid through medical insurance. The average total hospital expenses were 12520.54 ± 7844.88 ¥ (US$ 1929.20 ± 1208.11). Because the total hospital expenses data were not normally distributed (Z = 0.162, *P* < 0.01), the nonparametric test (Spearman test for continuous variables and Mann–Whitney test for categorical variables) was used for correlation analysis. The following factors were significantly associated with total hospital expenses: type of disease (*P* < 0.001), medical insurance (*P* < 0.001), classes of surgery (*P* < 0.001), minimally invasive surgery (*P* = 0.026), day surgery (*P* < 0.001), general anesthesia (*P* < 0.001), age (*P* < 0.001), and LOS (*P* < 0.001) (Table 2).

### Prediction of the total hospital expenses of patients undergoing breast cancer surgery

Eight factors were entered into the MLR model, which revealed six factors to be significantly associated with total hospital expenses: age, LOS, type of disease, having medical insurance, minimally invasive surgery, and receiving general anesthesia. The model had a sensitivity of 85.90% and a specificity of 70.45% with 0.886 of AUC (SE = 0.013, P< 0.001, 95% CI = [0.860, 0.907]).

**Fig. 1:**
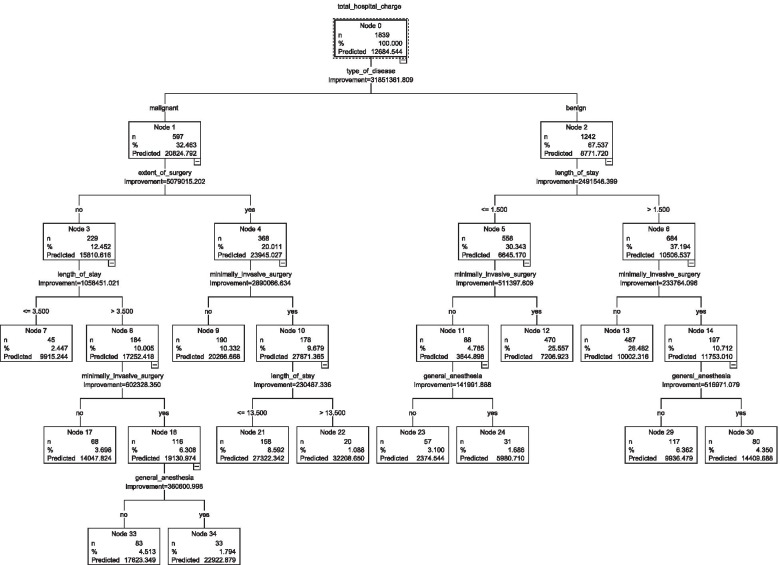
Regression tree of the CART model

The linear correlation in the CART model was 0.878 for the training dataset and 0.875 for the test dataset (Table 4). Sensitivity analysis (Table 5) revealed that the most critical variables for total hospitalization cost in the CART model were LOS (relative importance: 0.47), minimally invasive surgery (0.30), general anesthesia (0.19), age (0.02), and day surgery (0.02). The CART decision tree comprised 5 layers and 13 leaf nodes. The details of the classification rule are presented in Table 6 and Figure 1. The model had a sensitivity of 75.20% and a specificity of 71.39%, with 0.853 of AUC (SE = 0.014, P< 0.001, 95% CI = [0.831, 0.872]).

**Fig. 2:**
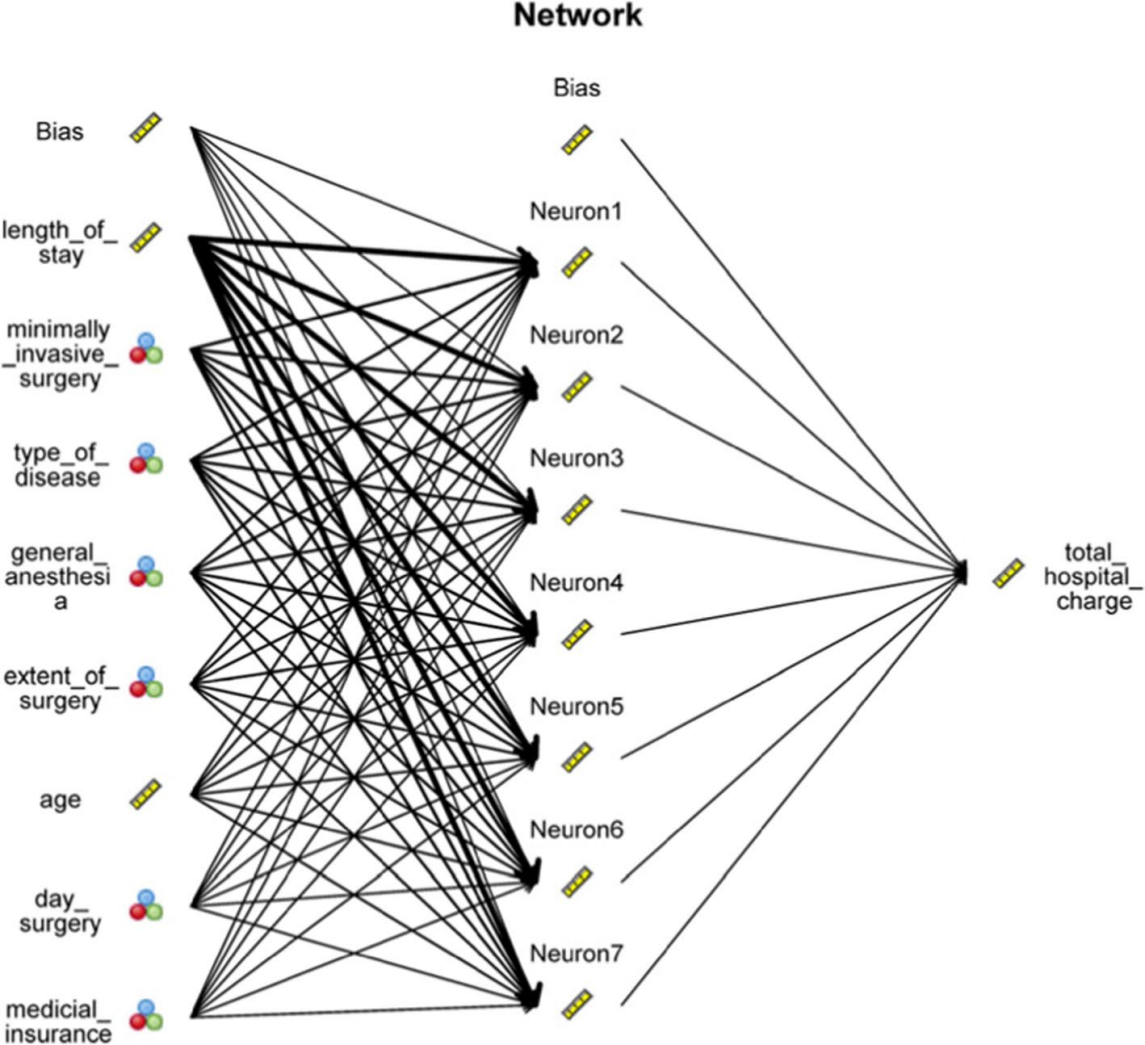
Topology architecture of the ANN model

The correlation coefficient in the ANN model was 0.884 for the training dataset and 0.880 for the test dataset (Table 3). Sensitivity analysis (Table 4) revealed that the most critical variables for total hospital expenses in the ANN model were LOS (relative importance: 0.48), minimally invasive surgery (0.14), type of disease (0.12), general anesthesia (0.09), classes of surgery (0.07), age (0.05), day surgery (0.02), and medical insurance (0.01). Figure 2 illustrates the topology architecture of the ANN model.

**Fig. 3:**
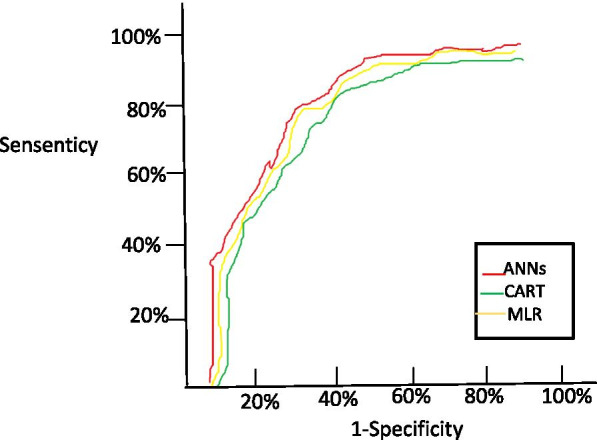
Comparison of AUC Between Three Models

Table 7 compares the performance indices of the three models, the comparison of ROC was presented in Fig.3. These models were significantly different from each other. (ANNs vs MLR, SE =0.003, *Z* =2.345, *P* =0.008; ANNs vs CART, SE =0.010, *Z* =2.598, *P*=0.001; MLR vs CART, SE =0.012, *Z* =2.210, *P* =0.01), which showed that ANNs was the best model to predict the total hospital expenses in patients undergoing breast cancer surgery. The sensitivity, specificity, PPV, NPV, FPR, FNR, F1-score , MCC and OA were 85.7%, 82.0 %, 86.3%, 81.7%, 16.2%, 13.4%, 82.34%,86.70% and 83.88,respectively, with 0.889 of AUC (SE = 0.012, P< 0.001, 95% CI = [0.860, 0.920]).

## Discussion

Surgery is one of the most effective treatments for diseases such as breast neoplasms, but it also involves economic consideration for patients undergoing breast cancer surgery. Total hospital expenses is a vital economic component of any hospital treatment, including breast cancer surgery. Its prediction is a common concern to patients, hospital managers, and medical insurance providers worldwide, for rising healthcare costs are a major public health issue. Thus, accurately predicting these costs including total hospital expenses and understanding which factors contribute to increases in total hospital expenses are important. It will both help patients save inpatient costs and hospitals reduce the medical care costs to promote single-disease or DRGs payment system based on the clinical information for China and other low- and middle-income countries. In this study, we used three models—MLR, CART and ANNs—and compared them to predict the main factors influencing the total hospital expenses of patients undergoing breast cancer surgery. The results indicated that LOS, major surgery or minimally invasive surgery, and general anesthesia were critical predictors of total hospital expenses.

Evidently, the longer the hospital stay, the higher the total hospital expenses [20–22]. In the present study, patients with longer LOS were usually those with clinically suspicious or scattered lesions who needed specific localization for benign diseases, whereas patients with malignant breast lesions with longer LOS were usually those who needed a comprehensive assessment of their overall condition, such as elderly women who tended to have multiple comorbidities or patients with locally advanced/metastatic breast cancer. Our results are consistent with several studies [23–25]. The variations in LOS for surgical patients might be attributed to either preoperative LOS or postoperative LOS [26]. Therefore, attention should be paid to not only the timely disposition of preoperative examinations and localization but also the treatment and improvement of patients’ comorbidities, such as heart disease and diabetes, before surgery, to minimize their influence on perioperative safety and postoperative rehabilitation. Accordingly, some targeted measures should be taken to reduce preoperative LOS by adding localization equipment, increasing the number of radiological technologists, and optimizing medical resource allocation. Postoperative LOS can be reduced by, for example, implementing a short-stay program after breast cancer surgery, which was considered feasible and safe [24]. In China, to shorten the LOS of patients with breast cancer, tertiary public hospitals should consider transferring patients to lower-level hospitals or primary health centers for further rehabilitation according to the guidelines on Chinese hierarchical medical service, thereby alleviating the patients’ economic burden and optimizing nationwide health care resource allocation.

However, taking account of the difficulty of dealing with the post-surgical complications from complex cases in low-level hospitals or primary healthcare centers, where postoperative patients with breast cancer were transferred to, some measures should be considered. On one hand, for tertiary hospitals where beds are limited, developing or improving the daycare system is a good option, for it can also achieve the goal of shortening the LOS and assure the safety of patients having surgical complications. In regards to this option, many prior studies have proved the role of daycare system on decreasing the LOS and financial burden for breast cancer patients [27–29]. On the other hand, for primary health centers with limited medical resources, developing a medical consortium united ward by integrating with tertiary hospitals may be another option. Through forming alliance with the trained surgeons from tertiary hospitals, having them reside in this united ward, and consistently educating them through routine diagnosis and treatment, the under-trained medical staff at grassroots-level institutions could gradually improve their capabilities of dealing with complicated cases and quality of healthcare services. In fact, following many developing countries, China has adopted the medical consortium united ward procedure as an alternative approach to treat transferred patients undergoing surgery [30–32].

Our findings also revealed that patients undergoing major or minimally invasive surgery were more likely to have higher total hospital expenses than those not undergoing these two types of surgery. To the best of our knowledge, major surgery is often connected with higher total hospital expenses in health economics. For patients with breast cancer, mastectomy is a common major surgery with serious risks and potential complications as well as higher total hospital expenses due to expensive surgical materials and complicated surgical process. These findings agreed with many previous studies [20–22, 33–36]. Differently, minimally invasive breast surgery is the preferred treatment for breast biopsies and some early-stage cancer treatments as keyhole surgery techniques advance. Early detection and treatment of breast abnormalities increase the chances of recovery with minimal disruption to the breast tissue. Compared with the traditional open surgery, minimally invasive breast surgery is generally clinically preferred to treat some breast tumors with a diameter of less than 3 cm, even if the location is relatively marginal, or the benign tumors have a diameter larger than 3 cm, minimally invasive breast surgery can also be considered. Minimally invasive breast surgery has the advantages including smaller surgical incision, less bleeding, quicker recovery with less pain, and lower postoperative infection rate. Moreover, adopting this surgery should consider whether it is combining with immediate reconstruction. Generally, immediate breast reconstruction is the first choice for early-stage cancer patients or those undergoing preventive breast resection. It is performed with mastectomy simultaneously, having features of not requiring secondary surgery, faster recovery and better anesthetics. Furthermore, if patients are at late-stage or have cancer metastasis, they have to select delayed breast reconstruction, which is usually performed when surgery and subsequent treatments are completed, in order to ensure a lower risk of complications and higher safety. There are some differences between two types of beast reconstruction on applicable conditions, disease risks, economic costs, anesthetic effects, and social psychology. However, in our study, patients undergoing minimally invasive surgery had higher total hospital expenses. This may be due to the relatively higher technical cost of minimally invasive surgery for breast cancer compared with normal open surgery, which is needed to achieve the dual goal of breast shape conservation and patient safety. This finding agrees with prior studies [34–36, 40]. Therefore, health policy decision makers should attempt to promote early detection and diagnosis of breast cancer and increase breast cancer awareness to facilitate the reduction of total hospital expenses and alleviate the economic burden for patients undergoing breast cancer surgery.

We also observed that receiving general anesthesia was a critical predictor of total hospital expenses in patients with both benign and malignant diseases. General anesthesia might lead to higher LOS and costs than other types of anesthesia [35, 36, 40].

Though receiving GA is more expensive and increases the hospitalization expense of patients with breast cancer, it is difficult to replace GA with non-general anesthesia in breast related procedures, due to the consideration that the regional and local anesthesia might cause a lot of psychology disturbance to the patients when patients have surgery on the breast under non general anesthesia. Some previous literature has reported the side-effects of using non-general anesthesia procedure for patients with breast cancer [41, 42]. If those patients were subjected to physiological and pathological changes, then affecting the patients’ vital signs (e.g. intraoperative heart rate, blood pressure stability) will be affected, which related to the success of the operation. Instead, choosing the short procedure in daycare setting might be a more relevant options rather than simply omitting GA. One feasible measure of shortening the procedure is integrating daycare surgery with the ERAS model. ERAS model is the integration of perioperative concepts using a series of tools proven effective by evidence-based medicine to intervene in perioperative patients to reduce surgical stress and complications, shorten hospital stays, reduce financial costs, and accelerate postoperative recovery [43–45]. It makes it possible to have breast cancer surgery in daycare setting, especially with technique of minimally invasive surgery. In turn, daycare surgery is also a concentrated expression of the ERAS model, which is widely advocated in the field of rehabilitation surgery. Among Shanghai’s tertiary hospitals, the ERAS is popularly implemented. This shortening procedure has been proved to have good results in orthopedics, including the clinical application in the field of artificial joint replacement, as it can reduce postoperative hospitalization time and mortality, increase patient satisfaction, and optimize medical resource [46, 47].

In addition, with the rapid development of anesthesia techniques, more economical techniques have been introduced. For example, thoracic paravertebral block (TPVB), as an alternative to general anesthesia, is being increasingly used in breast cancer surgery. TPVB can also be coupled with other regional anesthetic techniques, such as pectoral nerve block (PNB), thus reducing the LOS [48–50]. Thus, the application of TPVB or PNB might reduce the total hospital expenses. Nevertheless, further research is required to compare the cost-benefit and cost-effectiveness of the new anesthesia techniques and conduct an in-depth health economic evaluation of perioperative anesthesia programs for patients undergoing breast cancer surgery.

Notably, our data indicated that having medical insurance for patients undergoing breast cancer surgery was not as essential as expected in predicting the total hospital expenses in the mixed models. This indicated that cancer surgeons in the sample hospital strictly complied with the medical guidelines concerning cancer treatment and did not resort to overdiagnosis or overprescription based on the patients’ medical insurance status. Because China currently conducts a fee-for-service (FFS) payment method for medical service, which allows the clients to freely choose their physicians and hospitals, with very little interference from the insurance provider, doctors can order unnecessary tests and procedures to generate more income, thus encouraging them to practice “defensive medicine,” especially for cancer treatment. In many countries, payment methods for health care are based on a Diagnostic Related Group (DRG) system [50–52]. For example, in Germany, reimbursement for breast cancer-specific surgical interventions is regulated by the DRG system. Its actual treatment of breast cancer surgery is standardized according to the operation and procedure classification system (OPS) 2012, which reflects the basic principle of allocation of health care resources of variations [41]. Therefore, considering the advantages of the new payment method, since 2009, the Chinese government started exploring the switching of the FFS to DRG system in public hospitals in pilot cities to control the rapidly increasing health expenditures on cancer surgery, which commonly occurs worldwide [52]; it then started using the DRG system nationwide in 2019. The DRG system can provide a relatively accurate cost reference for patients before treatment, guide rational medical treatment, and facilitate the nationwide implementation of the Chinese graded medical health system.

However, our study had some limitations. First, if the target variable is binary or nominal, the models can be compared with the accurate matrix generated by the data mining system. However, the target variables in this study were neither, precluding their comparison with such an accurate matrix. Second, the data resources were limited. The dataset from the first pages of MRs we used did not include some other clinical information related to the disease, such as use of systemic therapy, type of breast cancer, radiotherapy, presence of visceral/bone metastases, presence of comorbidities, or functional status before surgery which may be critical for predicting total hospital expenses; addition of these variables may enable us to build a more accurate prediction model. The reasons of inability to add those variables are due to the freedom of seeking health services without rigid referral mechanism, some patients did not continue their treatments or follow-ups in Renji Hospital after surgeries, because they might come from other provinces outside Shanghai. This had made post-operative follow-ups difficult for us to conduct, in order to obtain further information on the post-operative use of systemic therapy or radiotherapy, or the presence of visceral/bone metastases, presence of comorbidities. Prospectively, for future study design and purposes, we probably will employ different data collection approaches to obtain more important variables mentioned above. Finally, the data were taken from a tertiary hospital in Shanghai, and no interregional and interhospital comparisons were conducted for the convenience of data availability; however, this limits the representativeness of our findings. Thus, future prospective studies with multicenter sampling are required.

## Conclusion

Our data indicated that total hospital expenses for patients undergoing breast cancer surgery were influenced by LOS, major and minimally invasive surgeries, and general anesthesia. These patient groups should be focused on to reduce the overall health care expenses. This study is the first to construct multiple models to predict total hospital expenses of breast cancer surgery. Our results revealed that the ANNs model was the best to predict patients’ total hospital expenses related to breast cancer, with relatively high accuracy; This may reduce the information asymmetry between doctors and patients and provide more reliable cost reference for patients, hospital managers, and medical insurance providers ,and lead to the establishment of more reasonable and practical inpatient medical consumption standards and reimbursement standards for patients undergoing breast cancer surgery.

## Supplementary Information

**Additional file 1.**

## Data Availability

Datasets analyzed during the current study are not publicly available due to privacy and ethical concerns, but are available from the corresponding author upon reasonable request.

## Abbreviations

THE: total hospital expenses
PPV: positive predictive value
NPV: negative predictive value
FPR: false-positive rate
FNR: false-negative rate
OA: overall accuracy
FFS: fee-for-service
MLP: multilayer perceptron
MLR: multiple liner regression
ANN: artificial neural networks
CART: classification and regression tree
LOS: length of hospital stay
